# Healthcare worker attitudes on routine non-urological preoperative urine cultures: a qualitative assessment

**DOI:** 10.1017/ice.2024.85

**Published:** 2024-10

**Authors:** Julia E. Friberg Walhof, Marin L. Schweizer, Kalpana Gupta, Madisen Brown, Daniel Suh, Judith Strymish, William J. O’Brien, Jeffrey Chan, Kelly Miell, Vanessa Au, Barbara W. Trautner, Kimberly C. Dukes

**Affiliations:** 1 Center for Comprehensive Access and Delivery Research and Evaluation (CADRE), Iowa City VA Healthcare System, Iowa City, IA, USA; 2 William S. Middleton VA Hospital, Madison, WI, USA; 3 University of Wisconsin–Madison, Madison, WI, USA; 4 Division of Infectious Diseases, VA Boston Healthcare System, Boston, MA, USA; 5 Center for Healthcare Organization and Implementation Research (CHOIR), Boston Campus, VA Boston Healthcare System, Boston, MA, USA; 6 Department of Medicine, Boston University School of Medicine, Boston, MA, USA; 7 Office of Rural Health, Veterans Rural Health Resource Center, Iowa City VA Health Care System, Iowa City, IA, USA; 8 Center for Innovations in Quality, Effectiveness and Safety (IQuESt), Michael E. DeBakey Veterans Affairs Medical Center, Houston, TX, USA; 9 Department of Medicine, Section of Health Services Research, Baylor College of Medicine, Houston, TX, USA; 10 Division of General Internal Medicine, Carver College of Medicine, Iowa City, IA, USA

## Abstract

**Objective:**

Many preoperative urine cultures are of low value and may even lead to patient harms. This study sought to understand practices around ordering preoperative urine cultures and prescribing antibiotic treatment.

**Design:**

Open-ended, semi-structured qualitative interviews

**Setting:**

5 Veterans Affairs hospitals.

**Participants:**

Interviews with 14 surgeons (9 surgeons, 5 surgical leaders), 7 infectious disease physicians, 8 surgical advanced practice providers (APPs), 1 surgical nurse manager, 3 infectious disease pharmacists, 1 hospitalist, and 1 lab manager.

**Methods:**

We interviewed participants using a qualitative semi-structured interview guide. Collected data was coded inductively and with the Dual Process Model (DPM) using MAXQDA software. Data in the “Testing Decision-Making” code was further reviewed using the concept of perceived risk as a sensitizing concept.

**Results:**

We identified themes relating to surgeons’ concerns about de-implementing preoperative urine cultures to detect asymptomatic bacteriuria (ASB) in patients undergoing non-urological procedures: (1) anxiety and uncertainty surrounding missing infection signs spanned surgical specialties, (2) there were perceived risks of negative consequences associated with omitting urine cultures and treatment prior to specific procedure sites and types, and additionally, (3) participants suggested potential routes for adjusting these perceived risks to facilitate de-implementation acceptance. Notably, participants suggested that leadership support and peer engagement could help improve surgeon buy-in.

**Conclusions:**

Concerns about perceived risks sometimes outweigh the evidence against routine preoperative urine cultures to detect ASB. Evidence from trusted peers may improve openness to de-implementing preoperative urine cultures.

## Introduction

Multidrug resistant organisms (MDROs) cause increased morbidity and mortality in American hospitals, resulting in more than 35,000 deaths annually.^1^ Over-ordering of urine cultures (UC) and subsequent overtreatment of asymptomatic bacteriuria (ASB) are major contributors to antibiotic overuse, which leads to antibiotic resistance.^2,3^ Recent studies show that urine cultures and antibiotic treatment of ASB do not prevent postoperative infections or surgical site infections.^4,5^ These unnecessary courses of antibiotics place patients at increased risk for antibiotic-related complications such as adverse drug reactions, *Clostridioides difficile* infections, and MDRO infections.^6–8^ The most recent Infectious Diseases Society of America (IDSA) Clinical Practice guideline (2019) recommends against screening for ASB in non-urological surgeries.^9,10^ Prior to this guideline, antibiotic treatment rates of detected ASB were almost 50%,^10^ and resistance to stopping urine cultures to detect ASB remains.^11,12^ While guidelines are necessary, targeted de-implementation strategies are often needed to create change in actual clinical practice.^13,14^ Recent data have shown that the guidelines alone have not substantially decreased rates of preoperative urine culture.^15–17^

The Dual Process Model of Cognition (DPM) has been used to better understand clinician decision-making processes.^18^ Helfrich et al.’s de-implementation model, which is based on the DPM, states that clinician decision-making is at least in part reactive to new information, which could result in rejection of new interventions if information is not viewed as coming from a familiar or reputable source, or if they feel their decision-making ability and professional authority has been curtailed.^18^ Helfrich et al.^18^ suggest unlearning through engagement with peers and substitution of new practices as potential methods to counter negative clinician reactance. Other studies identifying surgeon decision-making behavior related to perceived risk found that surgeons relied heavily on intuitive decision-making processes,^19^ and that those processes are driven by risk perception^20^ and available surgical options.^21^

Perceived risk is defined as the combination of uncertainty and potential severity of any related outcomes.^22^ Previous research has examined how clinicians assess potential risks and harms when making preoperative patient care decisions.^19–25^ Some of this work found that surgeons’ levels of risk aversion varied on an individual basis,^19,22^ and was based on perceived environmental threats as well as likelihood and severity of potential consequences.^19^ However, surgeons did not always have appropriate risk literacy to facilitate correct perception of risk likelihood and severity.^23,24^

Our study sought to gather information on what drives clinician decision-making regarding routine preoperative urine cultures. We aimed to inform future interventions to address practitioner needs and concerns when introducing new guidelines about preoperative urine cultures. We used perceived risk in the context of the DPM as a framing device to gain insight into how surgeons think about ordering urine cultures preoperatively, and we explored which perceived risks may keep them from de-implementing urine cultures for asymptomatic patients who are not undergoing genitourinary surgeries. This study was approved by the VA Central Institutional Review Board.

## Methods

We conducted 33 qualitative, semi-structured interviews with 35 participants across 5 Veterans Affairs (VA) hospitals around the United States between October 2020 and April 2022 (Table 1). The research team asked surgical or infectious disease (ID) leaders if their facility was interested in this research, then asked for names of staff who were involved in preoperative urine culture processes. Potential interviewees were invited via email to confidential interviews alone or in groups. Semi-structured interview guides, tailored to participant roles, consisted of open-ended questions about a range of topics including familiarity with current ASB guidelines, current ordering practice, and the decision-making process for ordering routine preoperative urine cultures for non-urological procedures (see Supplemental Material).

**Table 1.** Number of participants by role

Role	Total
Surgeons and surgical leadership	14
ID/Antimicrobial stewardship	7
Surgical advanced practice providers	8
Nurse managers	1
Pharmacy	3
Other	2
**Total**	35

Four members of the research team developed a thematic codebook together using a combination of inductively developed codes (or topical categories) and the DPM.^18^ At least two members of the qualitative team coded each interview together using a “negotiated” or consensus coding approach.^26^ Differences were resolved by a third team member. Two members further reviewed the data under the “Testing Decision-Making” inductive code using the concept of perceived risk^19–24^ as a sensitizing concept^27^ to identify reasons why clinicians might be reluctant to de-implement preoperative urine cultures.

## Results

**Figure 1.**
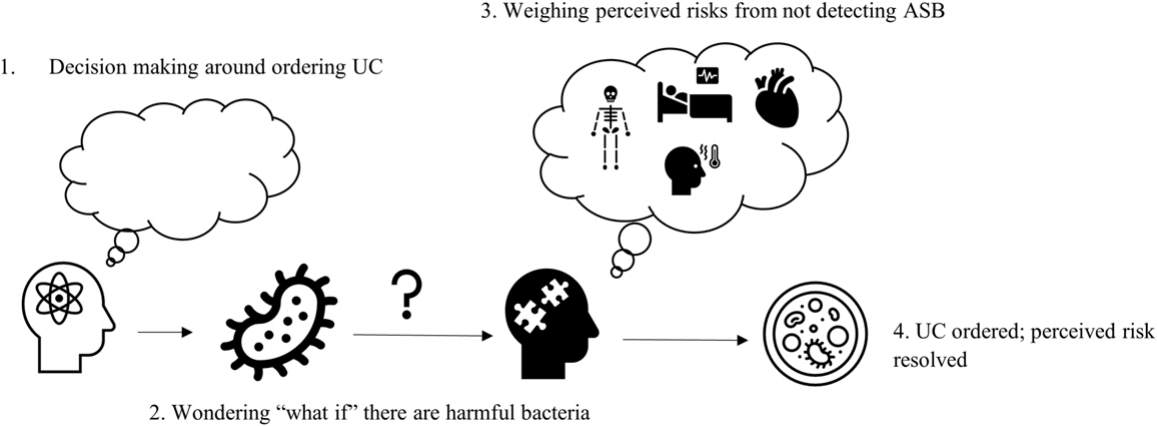
Impact of ASB risk perception by surgeons on preoperative UC ordering decisions. UC, urine cultures; ASB, asymptomatic bacteriuria.

We interviewed surgeons, infectious disease physicians, surgical advanced practice providers (APPs), a surgical nurse manager, infectious disease pharmacists, and others (Table 1). Participants had been in their current role for an average of 4 years, ranging from 1–20 years. We identified themes relating to surgeons’ concerns about de-implementing preoperative urine cultures to detect asymptomatic bacteriuria (ASB) in patients undergoing non-urological procedures: (1) anxiety and uncertainty surrounding missing infection signs spanned surgical specialties, (2) there were perceived risks of negative consequences associated with omitting urine cultures and treatment prior to specific procedure sites and types, and additionally, (3) participants suggested potential routes for adjusting these perceived risks to facilitate de-implementation acceptance. Examining surgeons’ risk perceptions in this way allows us to better understand surgeon rationale in continuing to order urine cultures to detect ASB, and what concerns should be considered when creating interventions to de-implement urine cultures (Figure 1).

### General perceived risk of infection through neglecting to order urine cultures

Participants reported variation in patterns of ordering urine cultures. Not all participants knew about the new guidelines. Participants suggested that for some surgeons, uncertainty around perceived risk that undetected and untreated ASB may cause postoperative complications was a significant factor in choosing to order preoperative urine cultures:

So this is where the fear comes among surgeon[s], if there’s an ongoing infection and it’s not detected, it’s very likely that the patient will have a complication or a post-op infection afterwards. (General surgeon, site 1).

Similarly, a participant at another site described how testing was sometimes done to assuage the “what if” anxiety that there might be a pathogen present that could cause problems postoperatively (Nurse practitioner 2, site 4).

For some participants, patient factors contributed to clinical decision-making about ordering preoperative urine cultures. They felt they could potentially prevent postoperative complications through antibiotic treatment. They described how there was some reluctance to test only symptomatic patients because they felt that Veterans might not experience symptoms the same way as non-Veterans (Table 2). Participants worried that symptoms of infection, or lack thereof, could also be highly dependent on individual patient health and on conditions such as a spinal cord injury or catheter use. These conditions can affect infection signs, and clinicians did not want to miss infections with potentially serious consequences. However, this point of view was not necessarily universal. Not all surgeons engaged in this practice. Some were individually less inclined, while some worked at institutions that had changed their policies. A few other participants emphasized that the worries expressed by their colleagues were not supported by current empirical evidence (Table 2).

**Table 2.** Key themes and exemplar quotes

Theme	Quote	Participant
General perceived risks of forgoing preoperative urine cultures to detect ASB	“Oh, gosh, if that joint gets infected, am I gonna be liable for not taking the UA?”…I think it’s more of a reassurance on the surgical pre-op side.	Nurse practitioner 2, site 4
	I think that some of our patients may not have the same symptoms that, you know,… your average remotely healthy person would have. …[Contrarily] I think that some people might misconstrue some symptoms as a urinary tract infection as well.	Lab, site 5
	They believe that [an infection from ASB] could happen and they would like to avoid by treating the bacteria preoperatively and eliminate that possibility. Although like, we don’t have very good evidence supporting that hypothesis.	Infection prevention specialist 1, site 5
	Some of them are understandably concerned about anything that might kind of increase the risk of infection in their patients.…it can be an uphill battle to convince them that “hey, you know what? Looking in the urine is really not going to improve your outcomes.”	Antimicrobial stewardship specialist, site 4
	I think in those situations the practice is probably driven a lot by fear, and that can be hard to deal with.	Infectious disease specialist 2, site 5
	I’ve heard a lot when, for example, when guidance changes, is that the provider who is going to do the ordering or the preoperative order knows that the guidance has changed, but doesn’t feel like their attending or someone else has endorsed those guidance yet. So much has changed because they are reluctant to make that person upset.	Infectious disease specialist, site 2
	There are other procedures that kinda fall through the cracks…orthopedic [and] cardiac comes to mind, that do urine studies despite our recommendations. ….Literature doesn’t support that.	Pharmacist, site 5
Specific surgical site concerns	We really have a low threshold for treating it., The risk, you know, the risk when we’re making a sternotomy uh, we’re just extremely concerned about any sort of an infection. Any sort of a bacteremia that might be present. …At the time that we’re making this incision, um, the consequences of a sternal wound infection are dire. So we really basically are trying to prevent it at all cost.…	Cardiac surgeon, site 4
Participant suggestions for reducing perceived risk	[if we hear about new guidelines] if one of us becomes aware of it whether it be in attending a conference…we bring that information back to the group….We of course… have talked to the other specialties involved whether it be anesthesia or um, the surgical staff, and it’s usually pretty well accepted. Especially when we bring it from…, from a vetted source….……	Surgical nurse practitioner, site 2
	You try to reach out to the individual surgeons, gauge what they’re doing and um, involve them in the decision making. So that if there is some protocol which is going to be put in place at you know,… multiple sites, that all the surgeons involved feel like they’ve been involved in the decision making and they’re vested in optimizing those kinds of practices.	Neurosurgeon, site 1
	…You know, cause that—they clearly want feedback for this one particular patient. That’s why they’re sending the consult. And so taking that opportunity to do a little teaching…, uh and kind of nudging um, a lot of times is really useful…	Infectious disease specialist, site 4

According to some participants, surgeons’ perceptions of risk were informed by fear of serious patient consequences. In the words of one ID physician (Site 5), “the practice is probably driven a lot by fear, and that can be hard to deal with.” Participants suggested that the strong perceived risk can make it difficult to convince surgeons to change their practices on this issue, even when there is evidence available.

### Concerns related to specific surgical sites and procedures

Participants reported that surgeons’ perception of the risk of ASB becoming a serious postoperative complication were also heightened for specific surgical sites and procedures. Surgeons performing orthopedic and cardiac procedures were extremely cautious to avoid any perceived risks. An antibiotic stewardship pharmacist (Site 5) suggested that some procedures “fall through the cracks” in efforts to de-implement routine urine cultures: “orthopedic [and] cardiac comes to mind, that do urine studies despite our recommendations.” Similarly, a general surgeon at another site (Site 1) reported that cardiac and orthopedic surgeons “are extremely worried about having a baseline undetected infection that … might compromise the prosthesis and result in a long-term infection afterwards.” At one site, worry about untreated ASB leading to prosthetic joint infections had led orthopedic surgeons to order urine cultures to potentially identify ASB. An orthopedic surgeon explained the rationale for the former long-time practice of preoperative urine cultures:

…the primary reason to do all this testing …is we don’t wanna miss a true UTI [urinary tract infection] for fear of having an increased risk of infection in the hip or knee replacement that we’re going to do….[so] if that’s your concern, you should check everyone and treat a lot of people. (Orthopedic surgeon, site 5)

This surgeon described a more recent change at their site, which shifted to a system of treating only symptomatic UTIs, but this quote serves as an illustration of the perceived stakes of untreated ASB for orthopedic surgeons.

The perceived stakes were also high for surgeons performing cardiac procedures. At Site 4, a surgeon and nurse practitioner described how with regard to sternal operations, cardiovascular surgeons were particularly insistent on ordering urine cultures to detect ASB. For example, the cardiac surgeon explained:

We really have a low threshold for treating it. … we’re just extremely concerned about any sort of an infection. Any sort of a bacteremia that might be present. …the consequences of a sternal wound infection are dire. So we really basically are trying to prevent it at all cost.

The chief of surgery at the site concurred that cardiovascular surgeons there reportedly considered that complications such as aortic graft infection would be “a death sentence” and would take all measures including ordering urine cultures to detect ASBs to prevent the complications they associate with them. Other roles were overall more willing to forgo routine cultures, but not all surgeons were unwilling.

### Participant-suggested avenues for adjusting perceived risk of reducing unnecessary urine cultures

To readjust this inflated perceived risk, participants provided suggestions for potential avenues of changing practice. Leadership support for reducing unnecessary urine cultures at multiple sites had helped create policy change. For example, one participant attributed the successful implementation of new policy around preoperative urine cultures to the arrival of a new chief of surgery and a new education lead, both of whom talked with and helped convince clinicians that the practice of ordering urine cultures to detect ASB was unnecessary and not evidence-based (Nurse practitioner, site 2). According to this participant, these conversations helped result in new policy where it had been stymied before.

However, at another site, a participant stated that an approach from the “top down” was possible, but not necessarily sufficient to create a lasting shift in perceived risk and unlearn practices. The surgeon suggested that surgeons may feel dictated to and resist changing their practice. Instead, the participant suggested taking a “bottom-up” approach to building support about practice change around ordering urine cultures among the surgeons involved:

...if there is some protocol which is going to be put in place…that all the surgeons involved feel like they’ve been involved in the decision making and they’re vested in optimizing those kinds of practices. (Neurosurgeon, site 4)

A similar suggestion came from a physician who was the antimicrobial stewardship champion at that same site, who suggested that ID specialists could provide education about guidelines during consults on patients.

Participants reported surgeons were generally reluctant to change preoperative urine culture practices, and suggested that it would take time and interventions on multiple levels to appropriately adjust risk perception and get them to accept new practices around urine cultures. Surgeons did not always trust evidence gathered by researchers. Endorsement from trusted colleagues was a factor in deciding whether to adhere to changing guidelines.

Surgeons, ID specialists, a hospitalist, and surgical APPs all suggested peer engagement as a potential strategy. This suggestion was made by interviewees both at sites that had implemented changes and at sites that had not implemented any changes in preoperative urine cultures. Helping surgeons unlearn their reactive practices to perceived risk can be a lengthy process according to participants, but participants also felt that support from colleagues and leadership could be a potential inroad to changing urine culture practice.

## Discussion

We found that some VA surgeons perceived that not ordering a preoperative urine culture is a serious potential risk for postoperative infection even when the patient has no symptoms of UTI. For some clinicians, this perception superseded current evidence that for most surgeries, cultures for ASB is unnecessary or even detrimental. Perceived risk of negative outcomes sometimes prevented surgeons from adhering to current guidelines. This risk was perceived as higher in some specialties, such as orthopedic and cardiovascular surgery. Participants provided us with salient suggestions for potential avenues for addressing these inflated perceptions of ASB in future interventions.

In 2019, new IDSA guidelines recommended against conducting urine cultures for non-urological procedures. This non-urological context had not been addressed in previous ASB policies.^28^ Despite this new evidence-based guidance, resistance to stopping routine urine cultures remains.^11,12^ Our findings support previous observations regarding reactive and perceived risk-driven surgeon decision-making, while also providing nuance on context-specific clinical judgments. Previous research found that while surgical decision-making varies on an individual basis, surgeons tend to rely on intuition, informed by perceived threats and consequences, when making clinical decisions to avoid perceived risk.^19–21,23–25^ Our findings focus these previous observations by providing specific context to inform the development of future interventions to de-implement preoperative urine culture ordering. The culture of each surgical department and its hospital is important to understand and may act as a contextual indicator of which approaches and interventions will be most acceptable to clinicians. Additionally, physicians at VA hospitals often also practice at nearby academic hospitals, which may impact their exposure and willingness to engage with new evidence and interventions.

In general, surgeons ordered preoperative urine cultures to detect ASB to reduce uncertainty around patient risks. Reasons given for wanting to order urine cultures included perceived risks related to ASB and potential surgical site infections for many surgeries, especially for cardiac and orthopedic surgery, concerns about the possibilities of missing infections that lead to surgical site infections, and wanting to avoid what they viewed as serious potential postoperative complications for their patients. However as previous studies describe,^23,24^ perceptions of infection risk among many surgical specialties was not always so accurate. Surgeons in our study made decisions reacting to these perceived risks. Some sought to spare their patients what they feared would be unnecessary potential morbidity and mortality. This fear could outweigh the evidence supporting de-implementing routine urine cultures. Since the guidelines did not specifically recommend against treatment of preoperative ASB for orthopedic implants, but rather focused on an earlier step of recommending not performing a urine culture, some clinicians feel obligated to treat once ASB is detected.

Leadership support and initiatives for de-implementation of routine urine cultures at three sites helped create policy change at those facilities prior to our study. However, participants at those sites suggested that peer engagement around the evidence could also be a useful form of engagement to improve surgeon buy-in for de-implementation. Engagement was suggested by participants at both sites that had de-implemented preoperative urine cultures, and at sites that had not. That this strategy was suggested by participants in different roles at non-de-implementing sites suggests that this is a strategy to which a wide range of clinicians would be receptive. These findings support previous studies’ conclusions that leadership support and stakeholder engagement were necessary to the success of de-implementation interventions,^29–32^ and that peer involvement from ID physicians could be important to facilitating de-implementation.^15^

Because the data in this study is qualitative, the sample size is relatively small compared to quantitative studies, and generalizability is limited. However, the depth of our sample (and diversity of stakeholders) adds strength to the robustness of the findings and utility in moving forward with future research focused on de-implementation.

Surgeons in our study made decisions around ordering urine cultures in reaction to perceived risks about missing ASB. Concerns about these perceived risks sometimes outweighed the current IDSA guidelines. Participants suggested that leadership support and peer engagement could improve surgeon buy-in. This potential strategy of peer involvement would fit well within the non-punitive focus for program adoption that is a part of the VA’s high reliability organization initiative.^33^ Future research should examine how interventions account for and address perceived risk in surgeons around de-implementing urine cultures, and what types of leadership or peer engagement could help improve acceptance of de-implementation initiatives regarding routine preoperative urine cultures.

## Supporting information

Friberg Walhof et al. supplementary material Friberg Walhof et al. supplementary material

## Supplementary material

The supplementary material for this article can be found at https://doi.org/10.1017/ice.2024.85.
