# Supplementary material for: Healthcare worker attitudes on routine non-urological preoperative urine cultures: a qualitative assessment
Source: Infect Control Hosp Epidemiol. 2024 Sep 19;45(10):1156–61. doi: 10.1017/ice.2024.85 (PMC11611506; doi:10.1017/ice.2024.85)
Supplement: Friberg Walhof et al. supplementary material [file S0899823X24000850sup001.docx]

**Example Interview Guide: Surgeons**

First, would you explain your role in the hospital? (Probe for types of surgeries, time at hospital, leadership roles)

**Current Practice**

Thank you. Next, I’d like to understand more about how pre-operative urine testing and antibiotic treatment work here. If you could, walk me through the process of making decisions about pre-operative urine testing for patients in your practice.

PROBING QUESTIONS:

- Is there a policy?
- Are decisions about ordering urine testing made by individual surgeons? Or by the head of the surgical group?
- Does anyone else at the hospital provide input about urine testing in your practice? (e.g., infection preventionists/stewardship team, lab, leadership, quality improvement/safety)
- What about on antibiotic treatment based on urine testing results?

What do you see as the main benefit of urine testing of pre-op patients?

Would you tell me more about what you consider as you’re making the decision about ordering urine testing for an individual pre-op patient?

PROBING QUESTIONS (if not introduced by interviewee):

- Could you tell me how you consider patient-level variables in this decision, if you do? (E.g., comorbidities, sex, BMI, race or ethnicity, ASA risk score, smoking status, fever, prior history of UTI or SSI, urinary catheter, mobility)
- What about the surgical procedure? (e.g., type of procedure, implants used, scheduled vs. urgent/emergent)
- In literature reviews and conversations we’ve had regarding urine testing prior to surgery, several factors have been discussed:
  - For example, concerns about infections and microorganisms carried by the patient and how they might impact their surgical outcomes. How do concerns about infection play into your decision, if they do?
  - When considering these potential infections, do you also consider potential antibiotic-related adverse events? (If yes) Could you tell me more about this? (If no) What are some of the main reasons you believe antibiotic-related adverse events are not considered in this process?
  - In other conversations we’ve had, medical liability is described as a concern. How do you think concerns about medical liability might play into your decision, if they do?
  - Any other concerns that we have not considered?
- In your practice, are there ever patients for whom you would not order urine testing?
- Are there patients for whom you would always order urine testing?

Could you explain what happens after you get urine testing results on a patient?

PROBING QUESTIONS:

- Do you get an alert if it’s positive?
- How do you make prescribing decisions based on the results?
- If the result is positive but the patient is asymptomatic, would you prescribe antibiotics?
  - Do you give up-front therapy or do you expand surgical prophylaxis?
  - Do you do a test of cure?
- Do you consult with ID in this situation?
- Would you feel comfortable not treating if ID (or other consultant) provided guidance that it was not indicated?

**Potential Readiness to Change**

Next, we wanted to explore whether and how new guidelines become part of routine practice. Recently released guidelines (Infectious Diseases Clinical Practice guideline on ASB) now recommend against screening or treating asymptomatic bacteriuria (ASB) for many surgeries. Often guidelines take quite a long time to be disseminated and integrated into regular practice. We’re interested in knowing how widely these new guidelines have been disseminated. Could you tell me if you are familiar with these new recommendations?

PROBING QUESTIONS:

- If YES, have the guidelines made an impact on your practice? Could you tell me more about this?

I wanted to follow up and have you think about the general process of addressing new guidelines and thinking about how, *or if,* to use them in practice. Could you walk me through what typically happens when you hear about new guidelines? How do you decide whether or not to incorporate new recommendations into your practice?

For you to consider any changes to pre-operative testing in your practice, what would you want to know about outcomes for patients?

- Are there certain groups of patients that you would be willing to try this for? Why or why not?

**Potential Interventions**

Since not all patients may benefit equally from urine testing and antibiotic treatment, we want to explore potential interventions to optimize urine testing, that is, to limit urine testing to only patients who will benefit. [For these questions, probe for more detail based on the description of practice process provided above.] Our project understands that providers have concerns (e.g., “like…” any introduced by interviewee earlier) and want the best outcomes for their patients. We are interested in your opinion on potential ways to address provider concerns and ensure urine testing is only used for patients who will benefit.

If surgical site infection is a concern, how would you feel about replacing urine testing and antibiotic treatment among patients with ASB with another type of testing and treatment?

To be more specific, one option might be nasal swabbing for MRSA or MSSA colonization, and antimicrobial treatment of nasal colonization. Do you feel that this is an option that would address concerns about surgical site infections? Could you tell me more about why?

When the laboratory has tried to limit the use of tests in the past, like the *C. difficile* test, they would require that a patient be symptomatic before the test was performed. Would a series of questions about the patient’s symptoms on the EHR prior to sending the test to the lab work in your practice? Why or why not?

Do you think audit and feedback about guideline concordance would work in your practice? Why or why not?

PROBING QUESTIONS:

- Is there anything that would help it work better?
- If YES, how would you appreciate getting that feedback? e.g., Would you want to see results at an individual level? What if you got more aggregated results? (e.g., at level of surgical service or hospital?)

Would you be interested in attending an interactive workshop to learn the current evidence about which patients may not benefit from urine testing? Why or why not?

Are there other ways you would suggest disseminating evidence about urine testing practices to providers? Do you have other suggestions that could help meet provider concerns about urine testing?

**Organizational Factors**

I’d like to ask a few broader questions about the institutional context in which you make decisions about urine testing and treatment.

*(Following questions will be asked in modified form depending on participant’s earlier responses.)*

Earlier you mentioned that infection prevention/the antibiotic stewardship team has/not provided input about urine testing and treatment. Do you see any role for the antibiotic stewardship team in conversations about pre-operative testing? What about in prescribing treatment based on these tests?

What about the lab? The quality improvement or patient safety team?

Optional depending on time:

- Has your practice tried any quality improvement initiatives about pre-operative urine testing? Would you tell me about that process?
- What about quality improvement initiatives to decrease treating positive urine culture results? Would you tell me about that process?

Do you have any experience with urine testing in non-VA hospitals? In your experience, is the culture of care around urine testing different in the VA than it is in other hospitals?

**Concluding**

Is there anything else we should be asking about pre-operative urine testing and antibiotic treatment?

Would you mind if I contacted you in the future if we have additional questions?

Is there anyone else you would recommend we talk to about urine testing?

Thank you so much for sharing your experience and perspective today.
